# From the migration crisis to the COVID‐19 pandemic, (im)possible regularization of migrants in Italy and Spain

**DOI:** 10.1111/imig.13069

**Published:** 2022-10-17

**Authors:** Juan Pablo Aris Escarcena

**Affiliations:** ^1^ Universidad de Sevilla Sevilla Spain

## Abstract

The reception of migrants, refugees and asylum seekers in camps has become a common phenomenon in Europe, discursively linked to the historical ‘crisis’ of mass movements towards the region. Camps and irregularity are two key issues in understanding the special impact that the COVID‐19 pandemic has had on migrants and refugees. This article explores connections between the ‘campization’ of migrant and refugee reception and the current debates for and against migrant regularization in response to the COVID‐19 pandemic in Southern Europe (Spain and Italy). The analysis uses qualitative methodology based on multi‐site ethnographic fieldwork (pre‐COVID‐19 pandemic); informal remote interviews with migrants and refugees; and analysis of political, media and legislative discourses.

## INTRODUCTION

During the so‐called ‘refugee crisis’ in Europe, camp‐like structures have become the main destination for exiles. This is evident in countries of first arrival, such as Greece and Italy, but it is also true for central European countries, such as Germany or France, where camps have reached the heart of Paris. Although a pre‐existing reality, camps in Europe have as a consequence of the ‘refugee crisis’ quickly been incorporated into the European imaginary.

When the precarious living conditions had disappeared from the European media and political agenda, the COVID‐19 crisis brought them back into the spotlight. The camps, naturalized as the place for ‘refugees’, reappear as the place of *exiles*, foreign citizens whose legal and administrative status is multiple and diverse but characterized by uncertainty and instability.^1^

The paper discusses the evolution of public policies of ‘regularization’, aimed at tackling growing exclusion in Spain and Italy. The first part establishes the theoretical and methodological framework for the analysis and the second part addresses public policies development, paying special attention to the importance of camps for their (in)viability. This paper aims to show how the camp, as a device and a place of governance, connects the effects that successive ‘crises’ have had in increasing social instability and uncertainty in migrants' lives, focusing on their legal and administrative dependency.

## ‘CAMPS’ AS A ‘FORM’ OF ANTHROPOLOGICAL LOCUS

The camps have generally been analysed from a negative perspective, influenced by the proposals of Agamben (1998) and his theorization of the camp as a place of exceptionality dominated by a sovereign power capable of ‘undressing’, of reducing the person to ‘bare life’. Agamben's ideas have been widely disseminated, as the analysis of necropolitics by Mbembé (2003) or the studies on extra‐territorial detention and ‘Graduated Sovereignty’ (Mountz, 2011; Ong, 2006). This current focus on Agamben's conceptualization of the mechanisms for radical exclusion from the political and social community as a core characteristic of Western tradition of government (which he traces back to Roman law) and its link to camps and encampments, as mechanisms for the production of that ‘nuda vida’ (bare life). The Italian philosopher links the mechanisms inherent to the lager with the mechanisms of the new camps emerging in today's world, going so far as to establish the camp as the destined form of the contemporary nation state, as its founding principle:

The camp as dislocating localization is the hidden matrix of the politics in which we are still living, (…). The camp is the fourth, inseparable element that has now added itself to – and so broken – the old trinity composed of the state, the nation (birth), and land. (…) The camp, which is now securely lodged within the city's interior, is the new biopolitical nomos of the planet. (1998: 99)

Any critique of this analytical approach must recognize its importance in highlighting the fractures in the application of formal equality of rights and the exercise of extreme forms of violence developing in the camp. G. Agamben made a great epistemological effort to link the philosophical tradition of M. Foucault on the mutation of powers and forms of domination, with the liberal philosophical tradition of H. Arendt on the refugee and the stateless persons as authentic subjects of universal human rights, and the problems arising from the imagined and practical overlap between human rights and citizens' rights. It must be acknowledged, moreover, that during the so‐called refugee crisis in Europe or the preceding crisis resulting from the wars in the former Yugoslavia, historical events seemed to corroborate this interpretation:

It is no coincidence that many former Nazi concentration camps in Europe were transformed into refugee “assembly centres” after the end of the Second World War and used again recently to host refugees during the recent “migration crisis” in Europe (on refugees in Dachau see Hardach, 2015; in Buchenvald see Huggler, 2015). (Martin et al., 2020: 13)

However, several authors have progressively challenged the interpretation of camps as places of bare life and exceptionality (or outright non‐lieux, as categorized by Augé, 1998). Largely influenced by the theories of Mezzadra (2006, 2011) and Mezzadra and Neilson (2013), a stream of critical analysts has gathered around the Autonomy of Migration paradigm (AoM) challenging Agamben's exceptionalist interpretation. In a U‐turn, the camps have come to be analysed from the point of view of their residents, as spaces of resistance, autonomy and the creation of a new kind of social and political belonging. An interpretation centred on the ‘migrant agency and citizenship created in motion’ (Rygiel, 2011). This analytical development has been carried out as a direct confrontation to interpretations centred on exceptionality:

…follows Agamben's theoretical insights on biopolitics (1995), one of the typical effects of humanitarian government is to reduce migrants and asylum seekers to “bare life”, that is, to a legally and politically disqualified form of existence (…) This reading rather underestimates the ability that migrants and refugees have to rebuild their own political agencies starting from the refugee camp, turning it into a scenario in which to put up a struggle, advance claims, and engage in conflict. (Campesi, 2015a, 2015b: 415–416)

Despite this, the critique fails to address the ‘negative’ character of the interpretative model of exceptionality. My analytical proposal is based on a different interpretation of M. Foucault's philosophical proposals, which focuses on the ‘positive’ or ‘productive’ dimension of the camp, understood as a device (a *dispositif*) and a place of power. I start from the research of Rahola (2003, 2007) who has developed his research focusing on: ‘The way in which they weigh on displaced individuals as a threatening possibility, the fact that they materially define the conditions of life in time (in terms of precariousness) and space (in terms of confinement) do not allow us to get rid of the camps as mere “‘lost” places’ (2007: 35).^2^ Thus, the camp does not apply an exception but is constituted as a sphere of production of new norms and forms of social belonging imagined by migrant collectives. It is characterized by its positive dimension, by the allocation of a physical and social space through techniques of knowledge–power and the exercise of power. The production of difference that operates through the camp is based on a series of interactions between a multiplicity of actors with clear disparities in their authority and forms of interaction (security actors, humanitarian actors, migrants, citizens), marked by power dynamics based on the processes of illegalization and criminalization of the presence of migrants with irregular administrative situations or precarious ‘regularities’ (Aris Escarcena, 2022).

The ‘camp form’ (Rahola 2003, 2007) is crucial in explaining how transnational migrants caught between diverse crises experience challenging and unstable policy contexts across Europe. F. Rahola defined the concept of ‘camp form’ as ‘a common matrix able to account for the various manifestations in which camps inhabit the present’ (Rahola, 2010).

In this paper, the term camp will be used to refer to very diverse types of structures, including reception centres, hotspots, transit centres, detention centres, self‐established camps and all the mixed forms of places of residence of migrants. The exercise of the above‐mentioned forms of power leads to the subaltern social incorporation of migrants into the territory and labour markets (Reigada Olaizola, 2022; Valluy, 2005); in fact, there is an interrelation between the prolonged presence of certain camps and forms of production based on intensive seasonal employment, labour and wage exploitation, informality and the frequent failure to comply with labour legislation. Examples can be found in the San Ferdinando camp (Calabria, Italy: Fekete, 2019; Peano, 2021), in Murcia (Spain: Gadea et al., 2015; Pedreño Cánovas, 1999) or in urban camps/camp‐like structures, formally designated as reception centres but which have in the wake of the ‘refugee crisis’ multiplied across Europe, from Athens to Calais (Ansems de Vries et al., 2016; Ansems de Vries & Guild, 2019).

## CAMPS IN 21ST CENTURY SOUTHERN EUROPE

The territoriality of the ‘new’ camps in Europe, partly as an exercise formally designed to govern a population and partly as an autonomous action of migrants or as part of broader migratory strategies, is conditioned by a series of social and economic dynamics, from the local context in which they are inserted to their connection with global networks of mobility. In this sense, the birth of camps inhabited by the illegalized migrant population is a process that is typical of our historical present, starting with the processes of illegalization of migrants in the 1980s and especially with ‘the end of the two blocs world’ or the ‘short 20th century’ (Hobsbawm, 1996). The territoriality of these camps (Agier, 2014; Picker & Pasquetti, 2015) is decisive, because one of the main characteristics of the camp as a device is the exercise of control techniques through the *milieu* (‘The space in which a series of uncertain elements unfold’, Foucault, 2009: 35). The physical separation of reception/detention structures from urban centres, or the connection of certain camps to large areas of agricultural exploitation, is the result of this interaction between the strategies of incorporation and settlement (sometimes of survival) of migrants and the dynamics of governance linked to what has been called by De Genova (2013) the spectacularization of illegality (‘the scene of exclusion, the obscene of inclusion’) or what I have named here ‘differential inclusion’ (Mezzadra & Neilson, 2013: 191. See also: Andrijasevic, 2009; Rygiel, 2011).

The first migrant detention centres in Europe came into being only 30 years ago, starting in the 1990s in the countries that pioneered the governance of migration through the contemporary paradigm of the ‘ir/regularity’ of migration, such as the United Kingdom (Bloch & Schuster, 2005). The first camps are situated in the same chronology, especially after the process of exile from the former Yugoslavia (Rahola, 2003), which involved human displacements towards other European countries, with larger numbers than the arrivals from the Middle East and Syria in particular during the ‘refugee crisis’. From these years onwards, some of the ‘camp‐form’ settlements were born, which would be key in the development of a whole genealogical series and European geography of camps linked to the governance of migration, such as the birth of the Sangatte camp (Fassin, 2011) and the genealogy of the camps (the so‐called jungles and squatter camps) in the Calais region (Aris Escarcena, 2019; Da Silva, 2014; Davies et al., 2017).

In southern European countries, the governance of migration through ‘irregularization’ is historically linked to their incorporation into the EU and their involvement in the common governance of external borders. In Italy, Spain and Greece, legislation was developed emulating northern States, based on prior contractual ties to obtain a visa and a work and residence permit. However, the insertion of migrants in these economies was very different from the United Kingdom or Germany, and they lacked the transnational recruitment networks that these had developed. The general tendency was to obtain regulation as a professional after a period of supervening irregularity: Migrants from these countries arrived on tourist or study visas, remained in the State after their visa had expired and subsequently obtained regulation either through ordinary provisions linked to their long stay and the offer of an employment contract, or through a massive process of ‘regulation’ or ‘amnesty’. The ‘shortcomings of the legal framework’ (as defined in the Spanish case by Solanes, 2010. See also: Chauvin & Garcés‐Mascareñas, 2012) cause migrants to live ‘incoherent’ situations, marked by ‘paradoxical real statuses’ (Jaegermann, 2011: 208). With the same approach, Düvell points out that the first contemporary migration laws came into force in the late 1980s in Spain (1986), and in the 1990s in Italy (1990) and Greece (1991) and were inspired by the integration of these countries into the EU: ‘Instead, before and after the introduction of immigration laws, immigrants usually obtained a legal status through posteriori “regularization” (Italy) or “normalization” programmes (Spain)’ (Düvell, 2011: 281). The same can be said of Greece, as pointed out by Apostolos (2018: 70): ‘in fact, Greek migration policy is, predominantly, a normalisation policy, that is, a policy of post‐hoc “legalization” of irregular immigration’.

The so‐called refugee crisis at the European level has had the effect of aggravating the precariousness of the status of migrants and the multiplication of camps. Defining this historical process as the will of governments to contain, fracture, disrupt and filter the movements of migrants is a simplification of a multifaceted process based on the rise of situations of vital uncertainty and differential inclusion, marked by the hegemony of detention and humanitarian reception as techniques for governing migrants and the camp as a device. A paradoxical consequence of the historical evolution of migration governance in Europe, culminating in the ‘refugee crisis’, has been the precariousness of the social and administrative recognition of exiles. The multiplication of camps is therefore parallel to the multiplication of statuses marked by irregularity or precarious regularity (such as that of people who receive humanitarian permits for 1–3 years, and have to renew them frequently).

Moreover, the ‘refugee crisis’ has contributed to the naturalization of the key elements of this social dynamic in the collective imagination of European citizens. Firstly, through the institutionalization of this type of device and its incorporation into EU normative sources, especially the development of the hotspots approach (Ansems de Vries & Guild, 2019; Avramopoulos, 2015; Garelli & Tazzioli, 2016; James, 2019; Papoutsi et al., 2018). Secondly, the narrative construction of the political crisis as a situation of emergency that ‘surprises’ governments, and of the humanitarian crisis as a situation of defencelessness and extreme vulnerability of migrants, have been the basis in the public debate to argue for this type of ‘liminal’ places, where a distinction is made between victims to be cared for/accommodated and ‘dangers’ to be contained/expelled.

Thus, while the refugee crisis had the effect of naturalizing the camps as a place where migration is governed, the crisis caused by the COVID‐19 pandemic has highlighted the unhealthy and precarious conditions in these camps, but also the uncertainty and instability of the migratory status of the exiles living in camps. One of the impacts of the COVID‐19 pandemic has been that many migrants have lost their residence or work permits (due to the loss of their jobs, or the impossibility of completing the necessary bureaucratic procedures) or have been unable to start the procedures to obtain these permits (either as a migrant or as an asylum seeker). Simultaneously, the pandemic has had a significant impact on the people living in the camps and camps‐like structures, although in disparate ways.

For exiles residing in shelters/detention centres, self‐isolation and social distancing may have been impossible to enforce and confinement to housing may have led to economic problems (Campesi, 2015a, 2018, 2020). Conversely, in camps in rural settings, economic activity has not stopped in the same way, the remoteness from any other inhabited nucleus and the limitations on mobility led to radical isolation and the impossibility of accessing certain social and welfare resources that migrants could count on (from visits to health centres or commercial areas, to contact with humanitarian or other organizations). Several exiles living in Borgo Mezzanone (a self‐made camp in Foggia, Italy) and other smaller camps in the region have expressed this concern: ‘They won't let us go to the hospital. We are only attended by the ambulance here in the camp, but if we go there they don't attend us…. even those who have the documents’ (Young man, Foggia). The ‘ambulance’ was, indeed, a mobile clinic operated by an NGO, which visited these camps once a week and had to deal with long lines of patients.

Exiles who were in the process of applying for asylum or subject to forms of administrative detention were crammed into places where the human right to health could not be guaranteed, such as the ‘quarantine ships’ in Italy^3^ (Farahat & Markard, 2020; Stierl & Dadusc, 2021) or ‘Arguineguín's dock’ (also called ‘camp of shame’) in Spain.^4^ One key case was the former camps and the new detention and first reception centres of the Canary Islands (Barbero, 2021). In October 2020, the judge Arcadio Díaz Tejera ordered the closing of the detention centre ‘Barranco Seco’, due to the mass infection of all exiles; however, the Spanish government's solution was to create two new ‘emergency centres’ to relocate the exiles:

The conditions were very bad, it was very hot and very cold at night… there were many sick people, with fever, and they were left in the room. When they closed the centre, they took my friend to the other island (Tenerife) to another place and he told me: “don't come, it's worse here”, so I escaped and slept on the beach… (Young man, Gran Canaria)

Those living in self‐established camps, with equally precarious living situations, were accused of being a vehicle of infection. Those in regular situations were affected by massive job losses during the crisis, and ‘coming back’ to the ‘camp’ was seen as a dramatic way out: either through detention or due to the need to seek income outside their previous occupation. In all these cases, migrants experienced immobility as a critical situation, blocking the precarious process of differential inclusion in European societies. At a medical visit to a small camp near Borgo Mezzanone, I witnessed an argument between a young resident and a medical care volunteer; the exile was tested as positive for Covid and they were discussing the resulting measures to take: ‘I am perfectly fine, I don't need the vaccine (the visit aimed at vaccinating the camp residents) or stay at home. I need to go to work tomorrow. I have only asked for a pill. If I stay at home, how I am going to pay the bills?’ (Young man, Foggia); the discussion became increasingly intense, as the volunteer pointed out that he was obliged to report his condition to the health authorities; taking advantage of the request to fetch his document, the exiled young man left the place and never came back.

The pandemic has led to a huge increase in the number of migrants in an irregular situation or legal limbo (e.g. asylum seekers unable to submit their applications, people unable to access migration offices to renew their permits etc.). This phenomenon has been read in many different ways (security, humanitarian, health, utilitarian), but it has caused a state of alarm in Europe. The responses have been diverse and marked by different national traditions, and the southern European states have reactivated the practice of ad hoc regularization. The cases of Italy and Spain are particularly significant, due to the structural characteristics that have been defined (limited possibilities of regular access, large economic sectors characterized by informality, historical application of massive regularization procedures etc.). Comparing the Italian and Spanish cases helps better understand the effects of migration governance in Europe.

## METHODOLOGY

The pandemic has transformed the way research is conducted (Fine & Abramson, 2020). In a context marked by unpredictability and by international and intra‐State mobility blockages, conducting ethnographic fieldwork has become a risky business. Throughout 2020, research has moved to the virtual environment. Digital ethnography, as a new methodology, allows the analysis of discourses and sociocultural processes through the virtual connection with groups and individuals. In contrast to classical ethnography, people no longer form stable groups or living communities; people who participate and express their ideas in groups or social networking platforms may reside in very distant places, maintain very different relationships in their local contexts and participate (or not) in multiple ways in civil society organizations. This multiplicity and interconnectedness of actors have the advantage of allowing social scientists to approach heterogeneous and complex collectives and interact with people who are highly motivated to discuss research topics (focus groups). The digital ethnography has been supported by analyses of legal and regulatory texts, as well as debates related to legislative processes.

However, migrant groups in situations of vulnerability have limited access to essential resources (internet, electricity etc.) to participate in these networks, especially irregular migrants, migrants with precarious and temporary administrative statuses and migrants living in ‘camps’. Migrants in more precarious situations do not tend to participate in political discussions in these social networks (with clear exceptions, as we will show below). In this unprecedented period, I have been able to conduct interviews with migrants I had met during my previous ethnographic fieldwork. Between 2016 and 2019, I conducted ethnographic fieldwork in several border areas of Europe, in Spain, Italy, France, Switzerland and Greece. For this research, I have resumed contact with migrants I already knew and many of whom, unfortunately, find themselves in the zone of precariousness and irregularity. In 2021, I was able to restart my fieldwork in Italy (in Puglia and Calabria) and Spain (in Andalusia and Canary Islands). I have conducted more than 30 formal interviews. And yet, my main approach in the fieldwork is informal chats, especially with exiles who are in a subaltern position to humanitarian workers (my main way to access the field is going with them) and who are ‘tired’ of being questioned, interviewed and interrogated by a panoply of police officers, humanitarian actors, journalists, health care professionals, bureaucrats and, also, researchers (Cabot, 2019).

This article will compare the cases of Spain and Italy, two similar processes in their structural characteristics (limited possibilities of regular access, large economic sectors characterized by informality, historical application of massive regularization procedures etc.) but with different developments, to better understand the effects that these crises have had on the consolidation of differential inclusion, and the importance of the accelerated dynamics of campization resulting from the ‘refugee crisis’ in times of pandemic. As explained below, one of the most relevant conclusions is that campization has been an obstacle to the administrative and effective inclusion of migrants in both Italy and Spain.

## ITALY, A REGULARIZATION THAT REACHES ‘NO ONE’

In Italy, regularization was proposed by the central government as part of the *Decreto Rilancio* (‘Decreto Legge n.34/2020, art. 103; 13 May 2020’), a set of measures to alleviate the social and economic effects of the pandemic and the confinement, covering a wide and diverse range of issues. The announcement of this regularization process was a crucial element of the decree. At the media level, the debate revolved around the emotional displays of the Minister of Agricultural Policies, Teresa Bellanova, when announcing this measure and her sensitivity towards the issue of ‘caporalato’ and migrants' claims. The discursive construction of this proposal included a utilitarian dimension (the need for a workforce) and also a humanitarian dimension, which emphasized the moral obligation towards those who had suffered some of the most serious consequences of the first wave of the pandemic.

However, little media attention was given to the content of this regularization proposal which was very restrictive. It focused on specific economic sectors that were of central importance in the redefinition of social and economic dynamics in the initial moments of the COVID‐19 pandemic: the agricultural sector; domestic work and care work (strongly interlinked).

This policy has been presented as an attempt by the government to ‘shed light on’ exiles working in the irregular economy, rhetoric already frequent at the beginning of the 21st century in both Italy and Spain (Barbero, 2010: 37–38), but which assumes different connotations in the context of the pandemic, as it recognizes the social relevance of migrants and facilitates their recruitment in the agro‐industry, domestic and care work sectors. In this sense, the regularization proposal is a ‘top‐down’ strategy.

The measure introduced by the *Decreto Rilancio* has had a very unequal impact on the domestic work sector and the care economy in comparison with agribusiness workers; but it also has a very unequal impact depending on the type of status it guarantees and promotes, with precarious‐temporary status taking precedence. The regularization measure had two channels: (1) The employer submits the regularization application based on an existing or new employment relationship (€500 for each application). A total of 207,000 applications have been submitted under this modality, of which more than 85% are for domestic and care workers; this means that only 15% are for dependent workers (mostly linked to the agricultural sector). (2) Workers apply to the prefectures for a temporary stay permit (at a similar cost) valid for 6 months, renewable with a formal work contract. Under this second modality, nearly 13,000 applications have been submitted. It is worth noting that of the total number of applications, some 220,000, only 14% have been examined (as denounced by the organizations involved in the campaign Ero Straniero).^5^ However, the preferred route has been the direct application by migrants (72% of the applications have been granted), that is, applications involving a precarious and temporary recognition (short term, 6 months); while applications submitted by employers have higher stability but have had a much lower acceptance rate, only 2.7% of the applications submitted have resulted in a residence and work permit, a total of 5600 permits granted.

In this chronicle of a failure foretold, the widespread use of the camp and the precarization of the status of migrants since the ‘refugee crisis’ has had a significant impact. It exists, mainly in southern Italy, where camp‐like ‘reception’ structures constitute places of high demand for precarious labour by the agri‐food industry. In the ‘piana of Gioia Tauro’ (a region in Calabria), around the town of Rosarno, the model of extensive agricultural exploitation based on the ‘caporalato’ has been nourished by migrant workers residing mainly in the so‐called ‘tendopoli’ (literally ‘city of tents’, used to refer self‐made camps in Italy) of San Ferdinando, born from a small self‐organized camp that grew exponentially since the middle years of the refugee crisis when the Italian government set up an extraordinary reception centre for asylum seekers. During my fieldwork, the migrants^6^ explained the practices of collusion between police, agricultural employers and street‐level bureaucrats (Lipsky, 1980). Many of the interviewees had been asylum seekers and had received humanitarian protection for 2 years; despite this, none of them had managed to formalize their employment relationship. This meant remaining in a situation of administrative instability and being dependent on precarious labour sectors:

the permit is useless. I can't go to France, I have to wait five years… and it doesn't help me to (get) a contract. It's very difficult… I tried once and my permit had already expired before I got all the papers (*laughs*). And it's all money, money, money, every single thing you have to do you have to pay for. (Young man, Foggia)

Most of the migrants I met in the *tendopoli* of San Ferdinando found themselves in a rat hole due to a combination of their administrative and socio‐economic situation: (1) They had no permit so it was very difficult to find formal work; (2) they were forced to work as day labourers in the crops of Calabria and Puglia; (3) they were integrated into an informal economy marked by the exploitation of labourers; (4) without ever having obtained a contract, and with a very limited income, they were not eligible for any regular status other than the renewal of their humanitarian permit (if applicable); (5) as confirmed to me by several informants, ‘the easiest way to renew the permit throughout Italy’ was to reside in San Ferdinando and go to Gioia Tauro to renew the permit, since the tents (even years after the camp was abandoned by the Italian authorities) were accepted as a domicile for this procedure. Migrants saw their situation limited to the necessary participation in a shadow economy (Reckinger, 2019) and to the residency in a place of humanitarian detention, to avoid falling into irregularity.

In a market based on irregularity and labour exploitation, the possibility of regularization has had a limited effect, because for many of the migrant workers involved in this industry, it was a possibility that had always existed and that employers had never wanted to exercise. One informant pointed out to me: ‘Here regularisation does not change anything. Because nobody is going to give you a contract. Even if you get a contract now, it's no good, because nobody has had a contract before’ (Young man, San Ferdinando). This significant second part shows that the only option for exile workers (living in camps) is the direct application for a permit, meaning self‐bearing the costs of the application and a 6‐month valid permit.

In this sense, the camps have become a reservoir of precarious or irregular migrant labour for the agricultural industry. The productive dimension of the camp as a governing device has been of central importance in the empirical configuration of these social dynamics, which we have called here differential inclusion. The pandemic has highlighted the relevance of these workers, but also the practical limits of a regularization measure that does not modify the dependence of migrants on employers, which has been at the heart of the demands for regularization in Spain, as presented below.

## SPAIN, SOCIAL MOVEMENTS FOR THE REGULARIZATION OF MIGRANTS

In Spain, unlike Italy, no regularization process has been approved for migrants who have fallen into irregularity. However, social and political mobilization around possible regularization has been much more intense than in Italy. In the Spanish case, the emergence of a platform connecting different migrant and human rights movements and organizations, which adopted the name ‘#RegularizaciónYa’ (#RegularisationNow), has been central. They were inspired by Portugal's strategy which, a few days after decreeing house arrest, announced that it would treat all migrants as citizens for a minimum period of 6 months. As the name itself shows, it is the result of a process of articulation through social networks and whose main activity was focused on communication and advocacy through them; #RegularizaciónYa was born as a social network hashtag, and its impact gave rise to an official platform linking different local and regional groups.

It is interesting to note the relevance acquired by migrants in terms of media and representation within the organization, something that is new in Spain. In addition to the relevance of some already consolidated migrant organizations, for example, the Popular Union of Street Vendors of Barcelona (Sindicato Popular de Vendedores Ambulantes de Barcelona) or ‘Top Manta’ union, several sectors that had no representation of their own or had very little visibility in the media and networks have gained great prominence. Two groups have stood out. On the one hand, domestic and care workers, who are undoubtedly the core of much of the platform's activity (Díaz Gorfinkiel & Elizalde‐San Miguel, 2021; Porras Vaca & Martín Pérez, 2020); they formed the Domestic Workers' Political Action Group (Grupo de Acción Política de las Trabajadoras de Hogar) linked to organizations such as the Association of Women Domestic and Care Workers (Asociación de Mujeres Empleadas de Hogar y Cuidados, SEDOAC, Madrid), the Union of Domestic and Care Workers (Sindicato de Trabajadoras del Hogar y Cuidados, Sindihogar/Sindillar, Barcelona) and the Association of Household Employees (Asociación de Empleadas y Empleados del Hogar, Seville). On the other hand, workers in the agri‐food industry have also been significantly involved in the platform. Normally these workers were represented by humanitarian or human rights organizations; however, a positive consequence of the pandemic has been the organization of agricultural workers and their connection with other groups.

The proposal launched by the ‘#RegularizaciónYa’ organizations clashed with the intention of the government which had developed a plan to promote the recruitment of foreign health workers already present in the territory and without authorization to exercise their profession, and of agricultural workers. Supported by the main governing party (PSOE) and the Minister of Inclusion, Social Security and Migration (José Luis Escrivá), it was a ‘concessionary’ proposal focused on addressing the needs generated by the pandemic. Its proposal discursively emulated European and global references (strongly connected), such as the ‘New Pact on Migration and Asylum’ and ‘The Global Compact for Safe, Orderly and Regular Migration (GCM)’ (Martín Díaz & Aris Escarcena, 2019), and in practice did not provide any possibility for the groups most affected by the irregularity and precariousness of migrant status.

Other political parties of the Spanish government, however, supported the proposal of the ‘#RegularizaciónYa’ platform, that is, a broader programme for the regularization of all migrants, which were presented by ERC (Esquerra Republicana de Catalunya) to the Spanish Parliament as a ‘Propuesta de No Ley’ (PNL).^7^ This proposal was characterized by its break with the traditional form of regularizations conditional on employment ties, and declared: ‘To establish a process of regularisation of a permanent nature for all persons currently residing in the Spanish State in an irregular manner, providing for the granting of an initial residence and work permit with special and simplified criteria for its renewal’. Unlike the Italian case, this proposal adopted a bottom‐up character: born out of the social movements led by migrants and supported by a large number of civil society organizations (more than a thousand joined the manifesto), the proposal took up the demands of migrant collectives.

In this process, the dual productive dimension of the camps is of enormous importance. Firstly, the articulation of new forms of political participation by illegalized migrants is generated in these places, from the Lucena camps in Huelva to the detention centres—the CIEs—where detainees have demonstrated during the pandemic. Very significantly, the growing relevance of migration as a central issue in public and political debate has opened up the possibility for these migrant groups to have significant relevance in the construction of the political agenda at the highest level (for a theoretical approach to political activism rising from camps: Frenzel et al., 2014). Thus, the camps are a point of political organization, despite being characterized as a governance device and a key place for the reproduction of the dynamics of differential inclusion. But it is this dual character that defines the camp as a productive place, a device that is a vehicle for the process of differential inclusion. During the pandemic and in the face of the refusal of the proposed regularization process, ‘camp‐form’ structures have been essential in guaranteeing the immobility and dependence of a pool of workers who have operated in even more precarious conditions than in previous years. The differential inclusion that occurs through the camp is at the legal, experiential, economic and social levels.

S.M., representative of the ‘#RegularizaciónYa’ platform in Seville and founder of an organization of migrant farm workers (Migrantes con voz propia/Migrants with a voice of their own), explicitly pointed to destitution as one of the main problems during the pandemic and how this allowed employers to tighten working conditions by dumping wages. He linked it directly to the ‘camp’:

In many places in Andalusia, it is incredible. We are hundreds of people working and the police and everybody knows it. If we stay in the fields (agricultural land) or the camps it's all right, but when we try to go to the villages… that's when the controls start, the detentions… I've had papers (work and residence permits) since before the pandemic, what I haven't been able to do is renew them, and even then it's very difficult for me to find someone from the village who wants to rent me a house or a room. So in the end you're waiting for the boss to want to rent you something, and often they rent you a bed in the field and ask you for a lot of money; but what are you going to do? Is it there or in the camp. During the pandemic, it was even worse, because many people who were working in (street) vending in the city came to the camp because they couldn't work in the city. And the bosses have taken advantage of this.

In fact, during the pandemic, job offers for agricultural workers have been detected in social networks, which openly established different wages depending on the administrative status of migrants. The expansion of ‘camp‐like’ housing structures that occurred during the ‘refugee crisis’, even if the increase in migratory flows across the Mediterranean only marginally affected Spain, is of enormous relevance in explaining the survival of certain socio‐economic dynamics even in times of pandemic and the instrumental nature of regularization proposals based on recruitment in the agro‐industrial sector. In the Spanish case, therefore, the camp is the place of production of the objective conditions of differential inclusion and, at the same time, of new forms of political participation and new forms of citizenship.

## CONCLUSIONS

Focusing on the paradoxical relationship between legal–administrative exclusion, the camp as a migration governance place and the differential inclusion at the socio‐economic level; the paper has analysed the policing efforts to face the consequences of the COVID‐19 pandemic. While the Italian government created a special administrative mechanism for regularization, in Spain, the parliament's majority rejected the ERC's legislative proposal. While the Italian regularization mechanism is a top‐down proposal with a markedly selective character (something that has determined its limited impact), the proposal born from social movements and migrant organizations in Spain has been innovative due to its inclusive character and the leadership of migrants in its drafting and advocacy. In both cases, the pandemic has highlighted the dependence of certain economic sectors (especially the agricultural industry and the care economy) and both states have large deficits in regular access channels. In both processes, the camp is a site of differential inclusion and, at the same time, a seedbed for new political initiatives and ways of imagining a common us.

The cases of Spain and Italy are relevant because they show just how difficult it is to untie this Gordian knot. The Italian government's utilitarian attempt at regularization failed to achieve its objective, as it was limited to a legal apprehension of the phenomenon and did not confront the social dynamics (caused partially by its own governmental praxis). The social movements' proposal for universal regularization in Spain did not achieve sufficient political backing, as it called for a profound change in the approaches that have defined contemporary migration policy.

Two main conclusions can be drawn about the role of camps in the cases of Italy and Spain: First, the camps generate a series of conditioning factors that hinder public policies such as attempts at regularization, but also others such as public health policies to control the pandemic (confinement, vaccination, tracing and monitoring of those infected). In this sense, the camp is a key dispositive in the reproduction of the dynamics of differential inclusion. It is not viable to have a policy that promotes encampments as a governance device and, at the same time, tackles the reproduction of pernicious dynamics such as labour informality, labour exploitation, lack of access to basic services, housing exclusion and other derived dynamics (such as collusion between law enforcement agents and actors in irregular markets).

Second, the humanitarian justification of the camps, the urgent need to protect vulnerable and endangered people, has been falsified. Many of the exiles living in these places have lived there since before the ‘refugee crisis’ and have continued to do so when the worst part of the pandemic seems to have passed. In Spain and Italy, governments have continued to use camps as the main device place for migration management. The official argument continued to be based on a humanitarian operational justification (and a humanitarian moral legitimacy), although health and social consequences showed that these policies were the cause of the main risks for exiles.

The pandemic has shown the complex grammar of camps, which are used in the production of new norms that limit the possibilities of exiles and increase the capacity of control (and even coercion) of certain actors over them, but also as a starting point for social networks, resistances and political projects seeking new alternatives for fairer incorporation into European societies.

### PEER REVIEW

The peer review history for this article is available at https://publons.com/publon/10.1111/imig.13069.

## Data Availability

Data sharing not applicable to this article as no datasets were generated or analysed during the current study.

## Endnotes

^1^
Throughout the paper, the term *exile/s* is used to refer without distinction to all those people who migrate seeking to settle in Europe and who do not have the possibility of doing so for a variety of reasons. It is an alternative to the technical terms of the legal–administrative categories (immigrant, asylum seeker, refugee etc.), which are very present in the public debate and are an essential component in the discourse and practices of government analysed in this paper.

^2^
Translated from French by the author.

^3^
The practice, as of April 2020, of confining migrants arriving across the central Mediterranean to Italy in large boats. A practice that has been defined as a ‘disturbing experiment’ and a ‘radicalisation of the hotspot approach’; see ASGI's latest report on this: https://inlimine.asgi.it/diritti‐in‐rotta‐lesperimento‐delle‐navi‐quarantena‐e‐le‐procedure‐di‐determinazione‐della‐condizione‐giuridica‐una‐radicalizzazione‐dellapproccio‐hotspot/.

^4^

https://www.eldiario.es/canariasahora/canarias‐opina/si‐previsible‐si‐habia‐alternativa_129_8703097.html & https://rtvc.es/se‐cumple‐un‐ano‐del‐llamado‐campamento‐de‐la‐verguenza‐en‐arguineguin/.

^5^

http://erostraniero.it/regolarizzazione2020/.

^6^
As part of the Documentary ‘Solidarity Crime. The borders of democracy’, some of the interviews with migrants in San Ferdinando are available on the project's youtube channel: https://www.youtube.com/channel/UCJkP0dKYjE6UoNIKIMa1sLg. If you would like to see the documentary or screen it at a seminar, please contact the production company *Mandorla Films* at the following address: contact@mandorla-films.com.

^7^
A lower level proposed law: ‘Proposición no de Ley presentada por los Grupos Parlamentarios Confederal de Unidas Podemos‐En Comú Podem‐Galicia en Común, Republicano, Plural y Euskal Herria Bildu, relativa a la regularización urgente, permanente y sin condiciones de todas las personas migrantes que residen en el Estado español’.
